# Investigation of Bacterial Infections Among Patients Treated With Umbilical Cord Blood–Derived Products Marketed as Stem Cell Therapies

**DOI:** 10.1001/jamanetworkopen.2021.28615

**Published:** 2021-10-07

**Authors:** Kathleen P. Hartnett, Krista M. Powell, Danielle Rankin, Paige Gable, Janice J. Kim, Samantha Spoto, Erin Breaker, Robert Hunter, Nychie Dotson, Gillian McAllister, Valerie Stevens, Alison Laufer Halpin, Hollis Houston, Erin Epson, Mary Malarkey, Melissa Mendoza, Lorrie McNeill, Kiran M. Perkins

**Affiliations:** 1 Division of Healthcare Quality Promotion, Centers for Disease Control and Prevention, Atlanta, Georgia; 2 Epidemic Intelligence Service, Centers for Disease Control and Prevention, Atlanta, Georgia; 3 Florida Department of Health, Tallahassee; 4 California Department of Public Health, Sacramento; 5 US Food and Drug Administration, Silver Spring, Maryland

## Abstract

**Importance:**

The number of clinics marketing stem cell products for joint diseases, chronic pain, and most recently, COVID-19, has increased despite warnings from the US Food and Drug Administration that stem cell products for these and other indications have not been proven safe or effective.

**Objective:**

To examine bacterial infections in 20 patients who received umbilical cord blood–derived products marketed as stem cell treatment.

**Design, Setting, and Participants:**

This case series is a national public health investigation including case-finding, medical record review and abstraction, and laboratory investigation, including sterility testing of products and whole-genome sequencing of patient and product isolates. Participants included patients who developed bacterial infections following administration of umbilical cord blood–derived products marketed as stem cell treatment during August 2017 to September 2018. Data analysis was performed from March 2019 to September 2021.

**Exposures:**

Umbilical cord blood–derived products marketed as stem cell treatment.

**Main Outcomes and Measures:**

Data were collected on patient infections and exposures. The Centers for Disease Control and Prevention performed sterility testing on undistributed and distributed vials of product marketed as stem cell treatment and performed whole-genome sequencing to compare patient and product bacterial isolates.

**Results:**

Culture-confirmed bacterial infections were identified in 20 patients (median [range] age, 63 [2-89] years; 13 male patients [65%]) from 8 US states who sought stem cell treatment for conditions including pain, osteoarthritis, rheumatoid arthritis, and injury; all but 1 required hospitalization. The most frequently isolated bacteria from patients with infections were common enteric species, including *Escherichia coli* (14 patients) and *Enterobacter cloacae* (7 patients). Of unopened, undistributed products sampled for testing, 65% (22 of 34 vials) were contaminated with at least 1 of 16 bacterial species, mostly enteric. A patient isolate from Arizona matched isolates obtained from products administered to patients in Florida, and patient isolates from Texas matched undistributed product sent from the company in California.

**Conclusions and Relevance:**

Unapproved stem cell products can expose patients to serious risks without proven benefit. Sequencing results suggest a common source of extensive contamination, likely occurring during the processing of cord blood into product. Patients and health care practitioners who are considering the use of unapproved products marketed as stem cell treatment should be aware of their unproven benefits and potential risks, including serious infections.

## Key Points

**Question:**

Were infections in patients who received umbilical cord blood products marketed as stem cell treatment associated with product contamination?

**Findings:**

In this case series, 20 patients in 8 states developed bacterial infections after receiving unapproved products marketed as treatment for conditions including chronic pain and degenerative joint conditions. This national investigation found widespread bacterial contamination of undistributed and distributed products from multiple donors, with whole-genome sequencing indicating a common source.

**Meaning:**

The findings from this outbreak underscore that unapproved and unproven stem cell products can expose patients to serious risks without clear benefit, including the possibility of product contamination.

## Teaser

This case series examines bacterial infections in 20 patients who received umbilical cord blood–derived products marketed as stem cell treatment.

## Introduction

Many clinics marketing stem cell products as treatment for joint diseases, sports injuries, and chronic pain opened in recent years^1,2^ despite warnings from the US Food and Drug Administration (FDA) that stem cell products for these and other indications have not been proven safe or effective.^3^ Recently, there has been an increase in marketing of unapproved stem cell products to prevent and treat COVID-19.^4,5^ The only FDA-approved stem cell products are derived from umbilical cord blood and are used to treat certain cancers and disorders of the hematopoietic system, such as leukemias, lymphomas, and sickle cell disease.^3,6,7^ The FDA has not approved stem cell products to treat any other diseases or conditions, such as osteoarthritis or aging. Generally, an unapproved stem cell product should be administered to patients only if there is an Investigational New Drug Application (IND) in effect for that product with FDA.

In September 2018, state health departments in Texas and Florida notified the Centers for Disease Control and Prevention (CDC) of bacterial infections in 7 patients (3 in Texas and 4 in Florida) who had received products derived from umbilical cord blood that were marketed as stem cell treatments; these were not FDA-approved or administered under an IND.^8^ The products had been processed by Genetech, Inc, and distributed by Liveyon, LLC, as the ReGen Series intended for treatment of conditions such as pain and arthritis. In response to reports of infections, Liveyon voluntarily recalled the ReGen Series products on September 28, 2018, and reported it would no longer purchase products from Genetech.^9^ On October 4, 2018, CDC issued a nationwide call for culture-confirmed infections in patients who received the ReGen Series products as part of a national investigation in collaboration with FDA and local and state public health departments, to better understand the causes of the contamination.

## Methods

### Epidemiological Investigation

CDC issued the nationwide call for infections through CDC’s Epidemic Information Exchange^10^ and provided health departments a standardized case abstraction form that captured information about patient demographic and clinical characteristics and product administration. Several health departments collected information from clinics on infection control practices through on-site observations or telephone interviews. Information about product manufacturing—from cord blood collection through distribution—was obtained through correspondence with Genetech and Liveyon representatives. Because this was an urgent public health investigation, CDC did not require institutional review board approval for this case series. Informed consent was not obtained because this was deemed an urgent public health investigation and the deidentified information was not used for research purposes, in accordance with 45 CFR §46. This study follows the reporting guideline for case series.

On December 20, 2018, FDA issued a warning letter to Genetech^11^ summarizing a June 2018 inspection of the processing facility that identified violations of manufacturing requirements, including insufficient donor testing and screening practices. In January 2019, on the basis of the results of the investigation, including new concerns regarding the potential risk of transmission of other communicable diseases (eg, HIV, hepatitis B virus, and hepatitis C virus), CDC recommended all patients who had received ReGen Series products be notified and counseled to consider testing for HIV, hepatitis B virus, and hepatitis C virus, and other infections as indicated.^12^ CDC distributed lists of health care practitioners who had purchased the ReGen Series products to state health departments, which worked with these practitioners to notify patients. To characterize the outbreak of bacterial infections, all infections found during the initial call for cases and patient notification were summarized as a case series.

### Laboratory Investigation

#### Sterility Testing

CDC received 3 types of product vials: (1) frozen, unopened, and undistributed vials from Liveyon’s headquarters in California that were in stock at the time of the recall; (2) thawed, unopened vials from Liveyon distributed to clinics and returned to the company after the recall; and (3) vials held by health care practitioners after patient infections. CDC performed modified sterility testing^13^ and species identification of detected microorganisms on all 3 types of product vials (see the eAppendix in the Supplement). CDC tested a sample of vials, prioritizing vials from cord blood donors whose blood was processed by Genetech within 14 days of the processing of a product vial associated with a patient infection; 3 vials per donor who met this criterion were selected. For the remaining donors, CDC tested 1 vial per donor. CDC quantified total microbial burden^13^ only in frozen, unopened and undistributed vials, on the basis of the assumption that burden of microorganisms, if present, would be higher in thawed, returned vials than in frozen ones.

#### Whole-Genome Sequencing and Bioinformatics Analyses

CDC also received 11 bacterial isolates (9 from 8 patients and 2 from products) from state public health departments. CDC used whole-genome sequencing (WGS) to compare the relatedness of patient and product isolates. CDC sequenced all patient isolates received. For products, CDC sequenced at least 1 representative isolate for every bacterial species identified from a vial that was also found in a patient isolate (see the eAppendix in the Supplement).

### Statistical Analysis

CDC collected the data using the REDCap electronic data capture tools (Project REDCap) hosted at CDC. We analyzed the data using SAS software version 9.4 (SAS Institute). Data were analyzed from March 2019 to September 2021.

## Results

### Epidemiological Investigation

As of March 2021, culture-confirmed bacterial infections had been identified in 20 patients (median [range] age, 63 [2-89] years; 13 male patients [65%]) following administration of these products for a variety of conditions, including pain, osteoarthritis, rheumatoid arthritis, injury, and quadriplegic limb spasticity. Infections totaled 8 in Texas, 4 in Florida, 3 in California, and 1 each in Arizona, Kansas, Maine, Colorado, and Massachusetts (Table 1). Five cases were identified when health departments contacted health care practitioners for the patient notification, and 2 others were reported following media coverage of the infections. Many of the remaining cases were identified by health care practitioners caring for patients with infections. Patients with infections received Liveyon products between August 2017 and September 2018, and most (14 patients [70%]) received the product between July and September 2018. Routes of administration included injections into patients’ knees, shoulders, spine, or digits; 2 received intravenous infusion, and 1 received a nasal spray. Ten patients had infections at the site of injection, such as septic arthritis of the injected joint or epidural abscesses, 5 had bloodstream infections, and 5 had both injection site and bloodstream infections. All but 1 of the 20 patients required hospitalization to treat the infection, with a median (range) duration of initial hospitalization of 9 (3-58) days. The most frequently isolated bacteria were *Escherichia coli* (14 patients) and *Enterobacter cloacae* (7 patients). Ten patients had polymicrobial infections. The 20 patients received products with 25 different lot numbers from at least 16 different cord blood donors.

**Table 1.** Demographic and Clinical Characteristics of Patients With Culture-Confirmed Bacterial Infections After Administration of ReGen Series Products From August 2017 to September 2018

Characteristic	Patients, No. (%) (N = 20)
Age, median (range), y^a^	63 (2-89)
Sex	
Male	13 (65)
Female	7 (35)
Route and site of administration^b^	
Intra-articular injection	16 (80)
Lumbar or cervical spine	8 (40)
Knee	7 (35)
Shoulder	4 (20)
Digits	1 (5)
Intradiscal injection	2 (10)
Intravenous infusion	2 (10)
Other^c^	2 (10)
Infection site^b^	
Bloodstream	10 (50)
Joint^d^	10 (50)
Spine^e^	4 (20)
Skin and soft tissue	2 (10)
Setting	
Pain clinic	7 (35)
Orthopedic clinic	6 (30)
Chiropractic clinic	2 (10)
Ambulatory surgery center	2 (10)
Spine treatment clinic	1 (5)
Medical spa	1 (5)
Rehabilitation and occupational medicine clinic	1 (5)
Patient’s reported reason for seeking stem cell treatment^b^	
Pain	12 (60)
Osteoarthritis	6 (30)
Rheumatoid arthritis	2 (10)
Injury	5 (25)
Other^f^	2 (10)
Unknown	1 (5)
Organism(s) isolated from cultures^b^	
* Escherichia coli*	14 (70)
*Enterobacter cloacae* complex	7 (35)
*Enterococcus faecalis*	6 (30)
*Citrobacter* species	3 (15)
*Proteus mirabilis*	1 (5)
Duration of initial hospitalization for infection, median (range), d^g^	9 (3-58)

^a^ Age was missing for 1 person.

^b^ Categories are not mutually exclusive.

^c^ Includes soft-tissue injection into the shoulder and nasal spray.

^d^ Includes osteomyelitis of the knee and septic arthritis of the knee and shoulder.

^e^ Includes epidural abscess, discitis, and vertebral osteomyelitis.

^f^ Includes shoulder adhesive capsulitis and quadriplegic limb spasticity.

^g^ Includes the 19 patients who were hospitalized. One did not require hospitalization.

Health departments in Florida and Texas investigated infection control practices at 5 clinics where patients received the ReGen Series product. One of the clinics may have used single-dose vials of saline for multiple patients, 2 clinics thawed the product vials in nongloved hands before administration, and no infection control breaches were reported from the other 2 clinics.

In correspondence with CDC, a Liveyon representative reported the umbilical cord blood used to produce the ReGen Series product was typically collected within the first few minutes after birth in a clean room and then sent to a blood bank for storage. The blood bank sent the cord blood to Genetech for processing, with the blood from 1 donor typically used to produce approximately 9 to 25 vials of product. Genetech reported that 10% of the product made from each donor’s cord blood underwent sterility testing. If no bacterial growth was detected after 14 days, all product derived from that donor’s cord blood was approved for distribution. Liveyon reported that it did not open vials it received from Genetech before placing each one inside a sealed pouch for distribution. Sealed pouches were then shipped to health care practitioners and clinics for administration to patients. An estimated 570 practitioners in 39 states and Puerto Rico purchased the ReGen Series product between the middle of 2017, when it was reportedly introduced to the market, and the September 2018 recall.

### Laboratory Investigation

CDC received 320 vials from Liveyon: 124 frozen, undistributed vials and 196 thawed, returned vials. The 124 frozen, undistributed vials included product derived from 17 umbilical cord blood donors. The 196 thawed, returned vials contained product derived from 64 umbilical cord blood donors. In addition, CDC received 12 distributed, unopened vials sent directly from clinicians in Texas (7 vials), Florida (4 vials), and Maine (1 vial). In total, CDC tested 160 vials: 34 of the 124 frozen, undistributed vials, 114 of 196 thawed, returned vials, and all 12 distributed vials sent to CDC from clinicians.

#### Sterility Testing

Overall, 86 of the 160 vials tested (54%) had bacterial growth, including 22 of 34 frozen, undistributed vials tested (65%), 54 of 114 thawed, returned vials tested (47%), and 10 of 12 vials sent by clinicians tested (83%) (Table 2). One of these vials was noted to have passed sterility testing in a report sent with the product from Liveyon to the clinic. A total of 16 bacterial species were identified, 4 of which were the same species as seen in patient infections (*Citrobacter freundii*,* E coli*,* E cloacae*, and *Enterococcus faecalis*). All other identified species were isolated from products only (Table 2). Among vials with bacterial growth, the most frequently identified species were *E cloacae* (41 vials [48%]), *E coli* (27 vials [32%]), and *C freundii* (26 vials [31%]). Ten of the 22 frozen, undistributed vials with bacterial growth contained enteric bacteria in excess of 10 000 colony-forming units/mL.

**Table 2.** ReGen Series Product Vials From Which an Organism Was Isolated During Sterility Testing at the Centers for Disease Control and Prevention

Organism^a^	Vials, No. (%)			
	Frozen undistributed vials (n = 34)	Thawed, distributed vials returned by clinics to Liveyon after recall (n = 114)	Vials held by clinicians (n = 12)	Total vials tested (N = 160)
*Enterobacter cloacae*	6 (18)	28 (25)	7 (58)	41 (26)
*Escherichia coli*	5 (15)	19 (17)	3 (25)	27 (17)
*Citrobacter freundii*	4 (12)	16 (14)	6 (50)	26 (16)
*Pseudomonas synthaxa*	6 (18)	0	0	6 (4)
*Stenotrophomonas maltophilia*	6 (18)	0	0	6 (4)
*Acinetobacter guillouiae*	4 (12)	1 (1)	0	5 (3)
*Citrobacter koseri*	0	5 (4)	0	5 (3)
*Staphylococcus epidermidis*	0	4 (4)	0	4 (3)
*Pseudomonas monteilii*	3 (9)	0	0	3 (2)
*Staphylococcus warneri*	0	2 (2)	0	2 (1)
*Enterococcus faecalis*	0	2 (2)	1 (8)	3 (2)
*Ralstonia pickettii*	2 (6)	0	0	2 (1)
*Staphylococcus pasteuri*	0	1 (1)	0	1 (1)
*Staphylococcus capitis*	1 (3)	0	0	1 (1)
*Staphylococcus saprophyticus*	0	1 (1)	0	1 (1)
*Acinetobacter calcoaceticus*	0	1 (1)	0	1 (1)

^a^ Organisms were isolated from 86 vials. Categories are not mutually exclusive because multiple species were identified in some vials. One vial had initial bacterial growth but did not continue, so a species could not be identified.

#### WGS and Bioinformatics Analyses

WGS was performed on 43 bacterial isolates: 9 isolates from 8 patients, including 6 patients in Texas, 1 in Arizona, and 1 in Colorado, and 34 isolates from products. See eFigure 1 and eFigure 2 in the Supplement for phylogenetic trees.

CDC sequenced a total of 16 *E cloacae* isolates (5 from patients in Texas and 11 from products). All 16 *E cloacae* isolates were highly related across different clinics and umbilical cord donors, differing by only 0 to 4 high-quality single-nucleotide variants (hqSNVs) across a core genome of 96.6%. The 5 patient isolates were highly related to 5 product isolates from frozen, undistributed products derived from 4 different cord blood donors, and to products held by clinics in 2 different Texas cities. Similarly, a *C freundii* isolate from a patient in Texas was highly related (0-4 hqSNVs across a core genome of 98.9%) to 9 *C freundii* product isolates (4 from undistributed vials and 5 from thawed, returned vials) derived from 4 donors.

CDC sequenced 13 *E coli* isolates (2 from patients and 11 from products). Four different multilocus sequence types were identified. Isolates differed between sequence types (21 747-34 604 hqSNVs across a core genome of 66.6%). However, isolates were closely related within sequence type (0-13 hqSNVs across a core genome of 96.16%-97.33%), including isolates from different donors, states, and sources, with an *E coli* isolate from a patient in Arizona highly related to 2 product isolates held by a clinic in Florida (0-1 hqSNV).

CDC sequenced 3 *E faecalis* product isolates. Two of these product isolates sent by the Florida clinic were from the same donor and were highly related to each other (0 hqSNVs across a core genome of 97.2%), but not related to a third product isolate from a thawed, returned product vial from a different donor (15 578 hqSNVs across a core genome of 82.5%).

## Discussion

In this case series, a national investigation identified 20 culture-confirmed bacterial infections in patients, most requiring hospitalization, who had received unapproved products derived from cord blood and marketed as stem cell treatment. The investigation found widespread contamination (65% of undistributed product from Liveyon in stock at the time of the recall and 47% of product that had already been shipped to clinics) that was not prevented by reported sterility testing. The contamination appears to have spread to vials from multiple donors, possibly during processing, according to WGS results that suggest a common source of contamination across products from multiple donors. Hundreds of clinics across the US unlawfully advertise stem cell treatments to patients as a cure for a variety of conditions for which there are currently no effective medical treatments, including certain neurological disorders, autism, and aging.^1^ Although some patients may be willing to risk unproven stem cell treatment over surgery for conditions such as chronic pain and degenerative joint diseases, this investigation demonstrates the potential risk associated with the use of these products.

Patients in this investigation most often had infections with *E coli* or *E cloacae*, common enteric bacteria that can colonize the gastrointestinal tract. These were also the most commonly identified bacteria in the products supplied to CDC for testing by Liveyon. If a health care practitioner does not use sterile technique during cord blood collection, enteric bacteria can contaminate the cord blood when it is collected around the time of birth. However, isolates obtained from products derived from multiple donors were matched by WGS, indicating that additional contamination or cross-contamination likely occurred after collection. FDA’s inspection found that Genetech did not adequately clean the processing environment or equipment between the manufacture of batches (ie, cord blood–derived products from different donors) and did not have any data or rationale for the cleaning agents used.^11^ The inspection also identified unlabeled product vials in the freezer awaiting sterility testing and lack of separation between products derived from different donors. Although this investigation could not rule out sporadic contamination at clinics, widespread contamination of unopened, never-distributed products sent directly from Liveyon to CDC and WGS results showing patient isolates matching product isolates from different states suggest that at least some contamination likely occurred before the vials were shipped to clinics. Further evidence supporting that some contamination occurred before vials were sent to individual clinics includes a patient isolate from Arizona matching isolates obtained from products administered to patients in Florida, patient isolates from Texas matching undistributed product sent from Liveyon in California, and the CDC finding widespread contamination in the frozen, unopened and undistributed, Liveyon-supplied product vials. Contamination at the time of product administration to patients cannot explain these laboratory findings.

Although the investigation found widespread bacterial contamination of the ReGen Series product, the investigation identified only 20 cases across 8 states. This might reflect underrecognition by both patients and practitioners, especially if the diagnosing practitioners had not been involved in product administration. In addition, reporting was voluntary and passive, because there is no requirement to report infections from unapproved products administered outside of a clinical trial. Practitioners who administered these unapproved products without an IND may feel a disincentive to report.

Unfortunately, the manufacturing practices that may have contributed to the contamination of the ReGen Series product are not unique to Genetech. After its recall of the ReGen Series products, Liveyon initiated production of a new umbilical cord blood–derived stem cell product in January 2019, and findings from an FDA inspection of Liveyon Labs, Inc, in May 2019 identified many deficiencies similar to those identified during the 2018 Genetech inspection.^14^ In March and August 2019, the FDA sent warning letters to 2 other firms processing umbilical cord blood into unapproved stem cell products, stating that they too failed to adequately prevent microbiological contamination.^15,16^ Patient safety requires strict adherence to infection control procedures for products at every stage, from collection^17^ and processing to administration, ensuring aseptic technique to prevent contamination of product and injection equipment.^18,19^

Some states and organizations have addressed the use of unapproved stem cell treatments. The state of California passed a law (SB 512) in 2017^20^ that requires health care practitioners offering unapproved stem cell products without an IND to post a notice for patients in a prominent location. The law also requires the Medical Board of California to compile statistics on complaints and disciplinary and administrative actions taken against licensees who provide stem cell therapies. In April 2018, the Federation of State Medical Boards adopted a policy for stem cell and regenerative treatments, including recommendations that state medical boards review marketing materials and claims, monitor FDA warning letters, and investigate unscrupulous or unprofessional practices.^21^

Although stem cells derived from umbilical cord blood have been shown to be effective for hematopoietic and immunological reconstitution in patients with disorders affecting the hematopoietic system, current evidence does not support the claim that stem cells are effective for other uses for which they are advertised, such as regenerating cartilage or repairing joints.^22^ Patients receiving these therapies for unapproved indications may do so out of a sense of desperation and in response to claims of benefit that might not be well supported. As patients make decisions about whether to receive unapproved stem cell products, it is important that they are aware of the potential risks associated with these products. Although Liveyon’s product was not approved by the FDA or administered under an IND, the instructions for use provided with the product to clinics claimed the product was “processed and distributed under the FDA requirements.”^23^

Manufacturers of unlicensed products, such as those associated with this outbreak, violate the law for profit at the expense of public health. Many create market confusion by erroneously describing their products as novel therapies that do not require FDA premarketing review or approval. That is not the case.^24^ Thus, FDA and CDC urge all patients and health care practitioners considering stem cell therapies to ensure that the stem cell product is being used for the approved indication or under IND and is on FDA’s list of approved products.^3,6^

### Limitations

This national investigation had limitations. First, most of the Liveyon-supplied vials tested by CDC were thawed, returned vials rather than frozen, undistributed vials (114 vs 34 of 160). The storage and handling of thawed, returned vials could not be ensured, so microbial detection could have been unrelated to collection or processing. Second, the investigation was unable to assess transport of product to Liveyon, storage of product at Liveyon, or distribution from Liveyon to clinics. Some contamination could have been introduced during administration at the health care facilities.

## Conclusions

The findings from this outbreak of 20 bacterial infections in 8 states underscore that unapproved and unproven stem cell products and treatments can expose patients to serious risks without proven benefit. Patients and health care practitioners who are considering unapproved stem cell products should be aware of the unproven benefits and potential risks of adverse outcomes, including serious infections.

## Notes

Supplement. **eAppendix.** Supplemental Methods **eFigure 1.** *Enterobacter cloacae* **eFigure 2.** *Escherichia coli*
eReferences

## References

[1] Knoepfler PS, Turner LG. The FDA and the US direct-to-consumer marketplace for stem cell interventions: a temporal analysis. Regen Med. 2018;13(1):19-27. doi:10.2217/rme-2017-0115 29327974

[2] Turner L, Knoepfler P. Selling stem cells in the USA: assessing the direct-to-consumer industry. Cell Stem Cell. 2016;19(2):154-157. doi:10.1016/j.stem.2016.06.007 27374789

[3] US Food and Drug Administration. FDA warns about stem cell therapies. Updated September 3, 2019. Accessed December 13, 2020. https://www.fda.gov/consumers/consumer-updates/fda-warns-about-stem-cell-therapies

[4] US Food and Drug Administration. Warning letters (filter to issuing office: Center for Biologics Evaluation and Research, letter issue date since March 2020). Updated March 9, 2021. Accessed March 11, 2021. https://www.fda.gov/inspections-compliance-enforcement-and-criminal-investigations/compliance-actions-and-activities/warning-letters

[5] US Food and Drug Administration. Enforcement Actions, CBER: BIMO/team biologics/internet surveillance/other. Updated February 22, 2021. Accessed March 11, 2021. https://www.fda.gov/vaccines-blood-biologics/enforcement-actions-cber/bimoteam-biologicsinternet-surveillanceother

[6] US Food and Drug Administration. Approved cellular and gene therapy products. Updated July 24, 2020. Accessed December 13, 2020. https://www.fda.gov/vaccines-blood-biologics/cellular-gene-therapy-products/approved-cellular-and-gene-therapy-products

[7] US Food and Drug Administration. Cord blood: what you need to know. Updated July 30, 2014. Accessed December 31, 2020. https://www.fda.gov/consumers/consumer-updates/cord-blood-what-you-need-know

[8] Perkins KM, Spoto S, Rankin DA, . Notes from the field: infections after receipt of bacterially contaminated umbilical cord blood-derived stem cell products for other than hematopoietic or immunologic reconstitution—United States, 2018. MMWR Morb Mortal Wkly Rep. 2018;67(50):1397-1399. doi:10.15585/mmwr.mm6750a5 30571672

[9] US Food and Drug Administration. Liveyon, LLC issues a voluntary nationwide recall of the ReGen Series^®^ product, Manufactured by Genetech, Inc. October 2018. Accessed May 2019. https://www.fda.gov/safety/recalls-market-withdrawals-safety-alerts/liveyon-llc-issues-voluntary-nationwide-recall-regen-series-r-product-manufactured-genetech-inc

[10] Epidemic Information Exchange (Epi-X). What is Epi-X? Updated April 4, 2018. Accessed December 13, 2020. https://emergency.cdc.gov/epix/index.asp

[11] US Food and Drug Administration. Warning letter: Genetech, Inc. MARCS-CMS 561808. November 29, 2018. Accessed December 13, 2020. https://www.fda.gov/inspections-compliance-enforcement-and-criminal-investigations/warning-letters/genetech-inc-561808-11292018

[12] Centers for Disease Control and Prevention. Stem cell and exosome products: warning about unapproved therapies. January 2019. Accessed June 2019. https://www.cdc.gov/hai/outbreaks/stem-cell-products.html

[13] US Pharmacopeia and National Formulary. USP 41-NF 36. 2016. Accessed September 1, 2021. https://online.uspnf.com/uspnf/document/GUID-AC788D41-90A2-4F36-A6E7-769954A9ED09_1_en-US

[14] US Food and Drug Administration. Warning letter: Liveyon Labs Inc. MARCS-CMS 588399. December 5, 2019. Accessed December 14, 2020. https://www.fda.gov/inspections-compliance-enforcement-and-criminal-investigations/warning-letters/liveyon-labs-inc-588399-12052019

[15] US Food and Drug Administration. Warning letter: Cord for Life, Inc. MARCS-CMS 572770. March 29, 2019. Accessed December 14, 2020. https://www.fda.gov/inspections-compliance-enforcement-and-criminal-investigations/warning-letters/cord-life-inc-572770-03292019

[16] US Food and Drug Administration. Warning letter: Stemell, Inc. MARCS-CMS 579013. August 28, 2019. Accessed December 14, 2020. https://www.fda.gov/inspections-compliance-enforcement-and-criminal-investigations/warning-letters/stemell-inc-579013-08282019

[17] The American College of Obstetricians and Gynecologists. ACOG committee opinion: umbilical cord blood banking. December 2015. Accessed July 21, 2021. https://www.acog.org/-/media/project/acog/acogorg/clinical/files/committee-opinion/articles/2019/03/umbilical-cord-blood-banking.pdf

[18] Centers for Disease Control and Prevention. Injection safety. Updated May 2, 2012. Accessed July 21, 2021. https://www.cdc.gov/injectionsafety/index.html

[19] Siegel JD, Rhinehart E, Jackson M, Chiarello L; Healthcare Infection Control Practices Advisory Committee. 2007 Guideline for isolation precautions: preventing transmission of infectious agents in healthcare settings. Updated July 2019. Accessed July 21, 2021. https://www.cdc.gov/infectioncontrol/guidelines/isolation/index.html

[20] California Legislative Information. SB-512. Health care practitioners: stem cell therapy. Accessed December 14, 2020. https://leginfo.legislature.ca.gov/faces/billNavClient.xhtml?bill_id=201720180SB512

[21] Federation of State Medical Boards. Regenerative and stem cell therapy practices: report and recommendations of the Workgroup to Study Regenerative and Stem Cell Therapy Practices; adopted as policy by the Federation of State Medical Boards. April 2018. Accessed December 14, 2020. https://www.fsmb.org/siteassets/advocacy/policies/fsmb-stem-cell-workgroup-report.pdf

[22] Marks PW, Witten CM, Califf RM. Clarifying stem-cell therapy’s benefits and risks. N Engl J Med. 2017;376(11):1007-1009. doi:10.1056/NEJMp1613723 27959704

[23] Liveyon. Instructions for use and product preparation guide: form 005v2 [package insert]. November 2017.

[24] US Food and Drug Administration. Regulatory considerations for human cells, tissues, and cellular and tissue-based products: minimal manipulation and homologous use. July 2020. Accessed July 21, 2021. https://www.fda.gov/regulatory-information/search-fda-guidance-documents/regulatory-considerations-human-cells-tissues-and-cellular-and-tissue-based-products-minimal

