# Supplementary material for: Investigation of Bacterial Infections Among Patients Treated With Umbilical Cord Blood–Derived Products Marketed as Stem Cell Therapies
Source: JAMA Netw Open. 2021 Oct 7;4(10):e2128615. doi: 10.1001/jamanetworkopen.2021.28615 (PMC8498849; doi:10.1001/jamanetworkopen.2021.28615)
Supplement: Supplement. — eAppendix. Supplemental Methods eFigure 1. Enterobacter cloacae eFigure 2. Escherichia coli eReferences [file jamanetwopen-e2128615-s001.pdf]

Supplemental Online Content

Hartnett KP, Powell KM, Rankin D, et al. Investigation of bacterial infections among patients treated with umbilical cord blood–derived products marketed as stem cell therapies. *JAMA Netw Open*. 2021;4(10):e2128615. doi:10.1001/jamanetworkopen.2021.28615

eAppendix. Supplemental Methods

eFigure 1. *Enterobacter cloacae*

eFigure 2. *Escherichia coli*

eReferences

This supplemental material has been provided by the authors to give readers additional information about their work.

eAppendix. Supplemental Methods

CDC's initial call was for any culture-confirmed infection in a patient who had received a ReGen Series product since February 1, 2018 (i.e., the earliest product administration date among patients recognized as of October 4, 2018). However, because CDC later received reports of infections in patients who had received ReGen Series products before this date, cases were ultimately defined as any culture-confirmed infection in patients who had received ReGen Series products since mid-2017, when Liveyon was believed to have begun distribution of the Genetech-processed products.

Health department staff either relied on practitioners involved in a patient's care to complete the case abstraction forms or completed abstraction forms themselves. Study data were collected and managed using REDCap electronic data capture tools hosted at CDC.[1,2] REDCap is a secure, web-based software platform designed to support data capture for research studies. Health departments submitted de-identified data to CDC via e-mail or directly uploaded information into REDCap.

Sterility testing

The outer surface of each vial was sanitized with 70% isopropanol before aliquots (0.25-0.5 mL) were injected into tryptic soy broth at a 1:10 dilution. The broth was incubated at 35°C for up to 14 days and screened for bacterial growth. Species identification was performed on all bacterial isolates using the matrix-assisted laser desorption ionization time-of-flight mass spectrometry (MALDI-TOF MS).

Undiluted product and serial microdilutions were placed on tryptic soy agar plates containing 5% sheep blood in duplicate and incubated at 35ºC for 48 hours before enumeration. When quantifying microbial burden, the lower limit of detection was 100 colony-forming units (CFU) per mL and the upper limit of detection was 300,000 CFU/mL.

Whole genome sequencing

Genomic DNA was extracted from bacterial isolates using the automated nucleic acid purification Maxwell 16 MDx system (Promega, Madison, WI), and DNA was sheared using the Covaris ME220 (Woburn, Massachusetts) to approximately 600 bp. Samples were then library prepped using the NuGEN Ovation Ultralow V2 System 1-96 kit and single indices (San Carlos, CA), and sequenced using the Illumina MiSeq reagent kit v2 (San Diego, CA) yielding 250 bp paired-end reads. Raw reads were processed using QuAISAR-H, a custom quality control and analyses bioinformatics pipeline.[3] Species identification was performed using Average Nucleotide Identity (ANIm) and samples were checked for contamination using Kraken and Gottcha.[4,5] Assemblies were generated using SPAdes, classified using multilocus sequence typing (MLST), and annotated by Prokka.[6-8] High quality single nucleotide variants (hqSNV) were enumerated and cluster core genome calculated using the Single Nucleotide Variant Phylogenomics (SNVPhyl) pipeline.[9]

eFigures 1 and 2 show whole genome sequencing results. Phylogenetic hqSNV trees from clusters linked to ReGen Series products, across multiple donors and multiple states for 1) *Enterobacter cloacae* (0-4 hqSNVs across a core genome of 96.6%) and, 2) *Escherichia coli (*0-34,604 hqSNVs across a 66.6% core genome). Phylogenetic hqSNV trees for each Genus display overall clustering as well as relatedness by branch length. Isolates on the same branch of the tree are more closely related to each other than to isolates on different branches.

© 2021 Hartnett KP et al. *JAMA Network Open.*

eFigure 1: *Enterobacter cloacae*

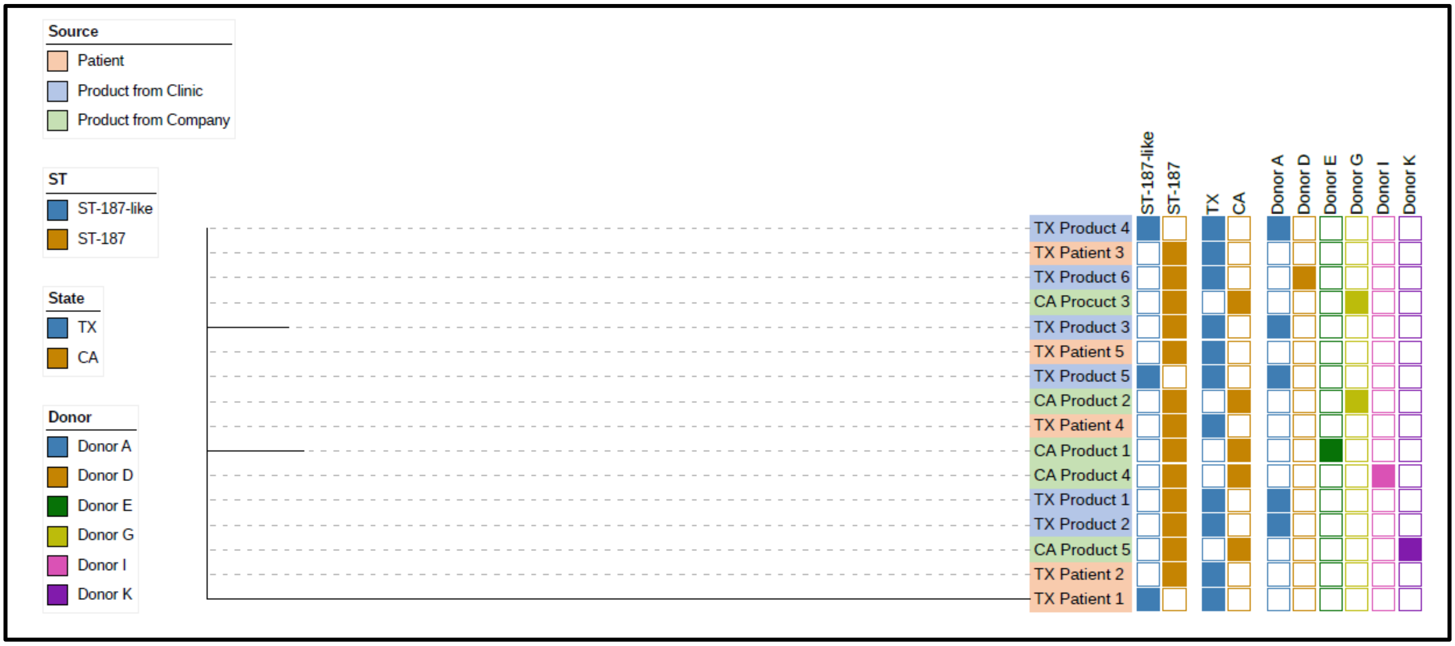

eFigure 2: *Escherichia coli*

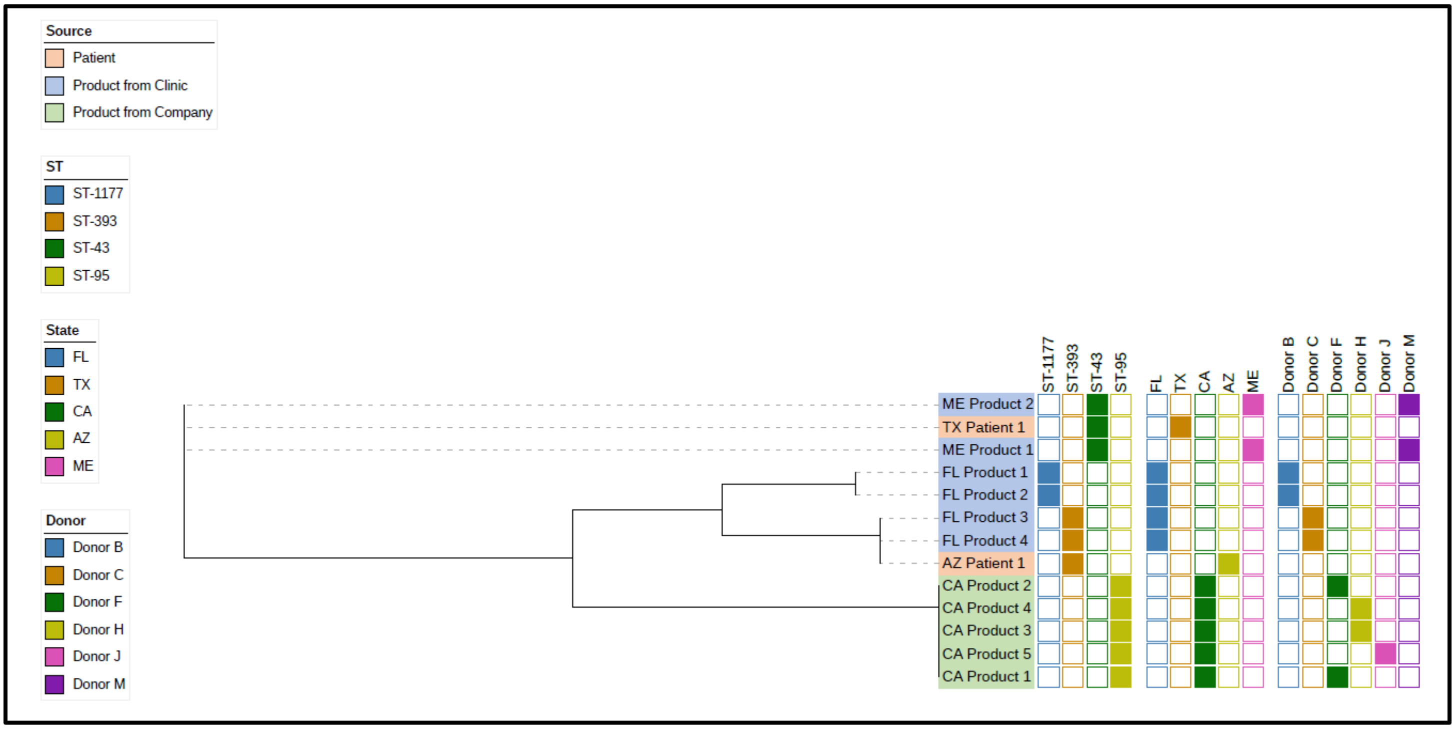

© 2021 Hartnett KP et al. *JAMA Network Open.*

eReferences

1. Harris PA, Taylor R, Thielke R, Payne J, Gonzalez N, Conde JG. Research electronic data capture (REDCap) – A metadata-driven methodology and workflow process for providing translational research informatics support. *J Biomed Inform. 2009 Apr;42(2):377-81.*
2. Harris PA, Taylor R, Minor BL, Elliott V, Fernandez M, O'Neal L, McLeod L, Delacqua G, Delacqua F, Kirby J, Duda SM, REDCap Consortium. The REDCap consortium: Building an international community of software partners. *J Biomed Inform. 2019 May 9 [doi: 10.1016/j.jbi.2019.103208].*
3. Stanton RA, Vlachos N, de Man TJB, Lawsin A, Halpin AL. Development and application of QuAISAR-H: A bioinformatics pipeline for short read sequences of healthcare-associated pathogens. ASM Conference on Rapid Applied Microbial Next Generation Sequencing and Bioinformatic Pipelines. 25 September 2018; Tyson Falls, VA.
4. Wood, D.E. and S.L. Salzberg, Kraken: Ultrafast Metagenomic Sequence Classification Using Exact Alignments. Genome Biology, 2014. 15(3): p. R46.
5. Freitas, T.A., et al., Accurate Read-Based Metagenome Characterization Using a Hierarchical Suite of Unique Signatures. Nucleic Acids Res, 2015. 43(10): p. e69.
6. Bankevich, A., et al., Spades: A New Genome Assembly Algorithm and Its Applications to Single-Cell Sequencing. Journal of Computational Biology, 2012. 19(5): p. 455-477.
7. Seemann, T., Mlst. Github https://github.com/tseemann/mlst.
8. Seemann, T., Prokka: Rapid Prokaryotic Genome Annotation. Bioinformatics, 2014. 30(14): p. 2068-2069.
9. Petkau, Aaron, et al. "SNVPhyl: a single nucleotide variant phylogenomics pipeline for microbial genomic epidemiology." Microbial genomics 3.6 (2017).

© 2021 Hartnett KP et al. *JAMA Network Open.*
